# Scaling analysis of field-tuned superconductor–insulator transition in two-dimensional tantalum thin films

**DOI:** 10.1038/srep42969

**Published:** 2017-02-20

**Authors:** Sungyu Park, Junghyun Shin, Eunseong Kim

**Affiliations:** 1 Center for Supersolid & Quantum Matter Research and Department of Physics, KAIST, Daejeon, 305-701, Republic of Korea

## Abstract

The superconductor–insulator (SI) transition in two-dimensional Ta thin films is investigated by controlling both film thickness and magnetic field. An intriguing metallic phase appears between a superconducting and an insulating phase within a range of film thickness and magnetic field. The temperature and electric field scaling analyses are performed to investigate the nature of the SI transition in the thickness-tuned metallic and superconducting samples. The critical exponents product of *νz* obtained from the temperature scaling analysis is found to be approximately 0.67 in the entire range of film thickness. On the other hand, an apparent discrepancy is measured in the product of *ν*(*z* + *1*) by the electric filed analysis. The product values are found to be about 1.37 for the superconducting films and about 1.86 for the metallic films respectively. We find that the discrepancy is the direct consequence of electron heating that introduces additional dissipation channels in the metallic Ta films.

The quantum phase transition^1^ of a two-dimensional disordered superconducting system can be achieved by both amplitude^2^,^3^ and phase fluctuation^4^,^5^ of the superconducting order parameter. According to the ‘dirty boson’^4^ model, the SI transition appears in the presence of the phase fluctuations induced by increasing either disorder or magnetic field. Within the framework, a superconducting phase corresponds to a vortex glass state in which the condensate of Cooper pairs appears with localized vortices, while an insulating phase corresponds to a Bose glass state in which Cooper pairs are localized by proliferated vortices. The scaling analysis demonstrates the existence of the universal critical resistance and find the critical exponents of *ν* ≥ 1 and *z* = 1 at the critical point^4^,^6^.

Recently, Ta^7^,^8^,^9^, MoGe^10^,^11^,^12^, exfoliated NbSe_2_^13^ and ion-gated ZrNCl^14^ systems exhibited an unexpected magnetic-field-induced intermediate phase between a superconducting and an insulating phase for a wide range of magnetic field. The intermediate phase is considered to be a metallic phase because of its finite saturated resistance at low temperatures. Besides, the current-voltage (IV) characteristics^7^,^13^ of the metallic phase showed remarkable distinction from other phases. The superfluid stiffness obtained by ac conductivity measurements^15^,^16^ demonstrates the possibilities of intermediated metallic phase as well. In addition to the magnetic-field-induced metallic phase, Ta^9^ and Bi^17^ thin films within a certain range of film thickness exhibit a disorder-induced metallic phase even at the zero-field and zero-temperature limit. A number of theoretical models were proposed to explain the unusual behavior^18^,^19^,^20^,^21^,^22^,^23^,^24^,^25^. However, the underlying physical mechanism that introduces the unexpected characteristics is not fully understood.

The scaling behavior of a two-dimensional disordered superconducting system is investigated in various materials^5^,^26^,^27^,^28^,^29^,^30^,^31^,^32^,^33^. Although the magnetic-field-induced SI scaling fails to obtain the universal critical resistance, the critical exponents show good agreement with theoretical predictions. Nevertheless, the scaling analysis of MoGe^26^ thin films shows notable deviation from the standard SI scaling behavior. The set of magneto-resistance curves collapses on the universal scaling function for high-temperature isotherms but exhibits remarkable deviation from the scaling curve for low-temperature isotherms. The product of critical exponents was found to be *νz*~1.3 at high temperatures, which was comparable to the that of InO_*x*_^5^ that exhibits the direct SI transition without any intervening phase. The apparent deviation of the low-temperature isotherm was attributed to the appearance of the magnetic-field-induced quantum metallic phase. One can find the observed deviation reflects essentially the low temperature saturation of magneto-resistance.

The critical exponents product of *νz* and *ν*(*z* + *1*) can be determined by scaling analysis with temperature and electric field, respectively. The correlation length exponent *ν* and dynamical critical exponent *z* can be independently determined by combination of these two products. However, one should be aware of a possible joule heating effect in the electric field scaling. The joule heating power, *P*, induced by applied current raises the temperature difference between the sample and electrons due to weak electron-phonon coupling at low temperatures. The electric field scaling fails unless 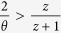, where *θ* is the exponent of electron temperature *T*_*e*_ to the power with the joule heating relation, 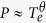. In other words, the electric field scaling is only valid when *θ* < 4 assuming that the dynamical critical exponent *z* is 1. The electric field scaling in MoGe thin films showed the product of *ν*(*z* + 1) ~ 2.65 and *θ ~ *4. The critical exponents show good agreement with theoretical predictions, although *θ* is found to be in the ‘marginal’ boundary. In addition, the electric field scaling analysis in the case of InO_*x*_ thin films^34^ was reported to be successful, although *θ* was about 6^35^. Nevertheless, the same scaling in InO_x_ failed with even smaller *θ* exponent of 5^36^. In addition, the electric field scaling in Bi thin films reported the product of *ν*(*z* + *1*) bigger than the expected value^37^. The authors suggested that the product *ν*(*z* + *1*) could be understood by transforming the electric field to the electron temperature, indicating the electric field scaling is equivalent to the temperature scaling. The excellent fit in the electric filed scaling can be thus attributed to the heating of electrons due to the weak electron-phonon coupling rather than the intrinsic electrical response.

In this paper, we report scaling analysis in Ta films with a wide range of film thicknesses that includes the disorder-induced metallic phase. Although scaling analysis is an excellent measure to identify the nature of a phase transition, no scaling study on the disorder-induced metallic phase has been reported. We found that the electric field scaling on very thin metallic samples was different from that of superconducting thin films. The combination of electric field and temperature scaling analysis shows that the apparent difference is the direct consequence of the electron heating that is more predominant in the thin metallic films.

## Results

**Figure 1:**
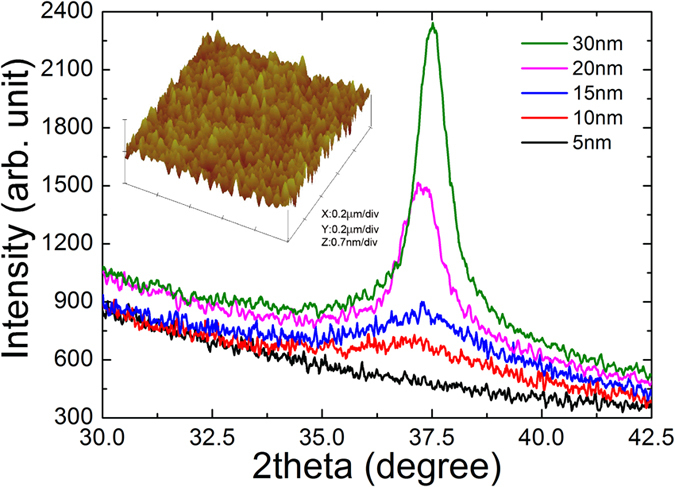
X-ray diffraction patterns of 5-, 10-, 15-, 20-, 30-nm-thick Ta films. Inset: Atomic force microscopy image of the 5-nm Ta thin film. The root mean square roughness is approximately 0.1 nm.

Ta thin films are characterized by X-ray diffraction (XRD) and atomic force microscope (AFM) measurements. Figure 1 shows the XRD results for Ta thin films with various thicknesses. Ta films with a thickness greater than 5 nm reveal a crystalline peak at ~37°, which is attributed to the local body-centered cubic (α-phase) correlation^38^. However, films with a thickness less than 5 nm do not show any peak structures in XRD, which indicates that these thin films are highly amorphous. An additional AFM image of the 5-nm Ta thin film is shown in the inset of Fig. 1. The root mean square (RMS) roughness is about 0.1 nm, which suggests that the film is spatially homogeneous. This roughness value is an order of magnitude smaller than the value for the InO_*x*_ thin films^39^.

**Figure 2:**
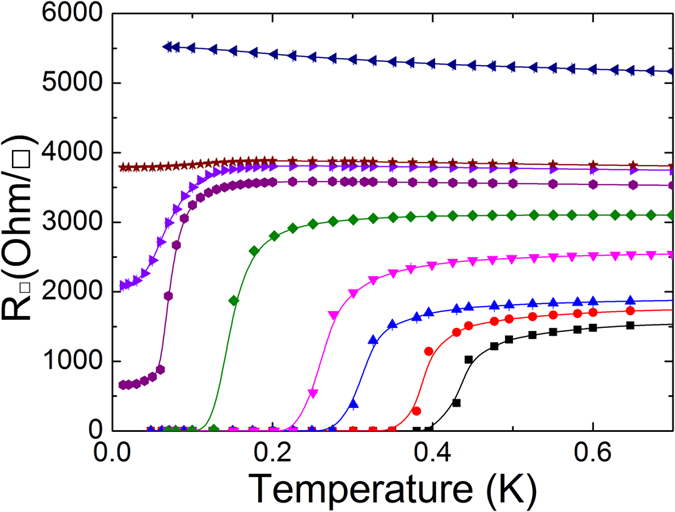
Temperature dependence of sheet resistance for Ta thin films of various thicknesses. The film thicknesses (from the bottom upward) are 5, 4.8, 4, 3.8, 3.6, 3.4, 3.35, 3.3 and 3.1 nm. The data points are raw data and the solid curves are guides to the eye.

The sheet resistance, *R*_□_, for various Ta film thicknesses is plotted as a function of temperature in the absence of magnetic field, as shown in Fig. 2. Superconducting thin films with a thickness between 3.6 nm and 5 nm show an abrupt resistance drop to zero at the critical temperature *T*_*c*_, and no evidence of re-entrant behavior is found. The critical temperature decreases with decreasing film thickness. This thickness dependence of *T*_*c*_ is the distinct feature observed in many other amorphous and homogeneous thin films^40^, while the *T*_*c*_ of granular superconducting films is independent of film thickness^3^. We observed the appearance of a disorder-induced phase that cannot be classified as either a superconducting or an insulating phase. Compared with the superconducting sample, the sheet resistance is saturated to the measurable finite value at the low-temperature limit. The broadening of transition is further enhanced, and the saturated final resistances at low temperatures increase with decreasing thickness, which is consistent with the previous observation^9^,^17^. Finally, further reduction of film thickness to 3.1 nm leads to a negative slope in d*R*/d*T*, which indicates that this Ta film is insulating. We find that these characteristic features of the disorder-tuned superconductor–metal–insulator transition are essentially the same as those of the previous studies^9^.

**Figure 3:**
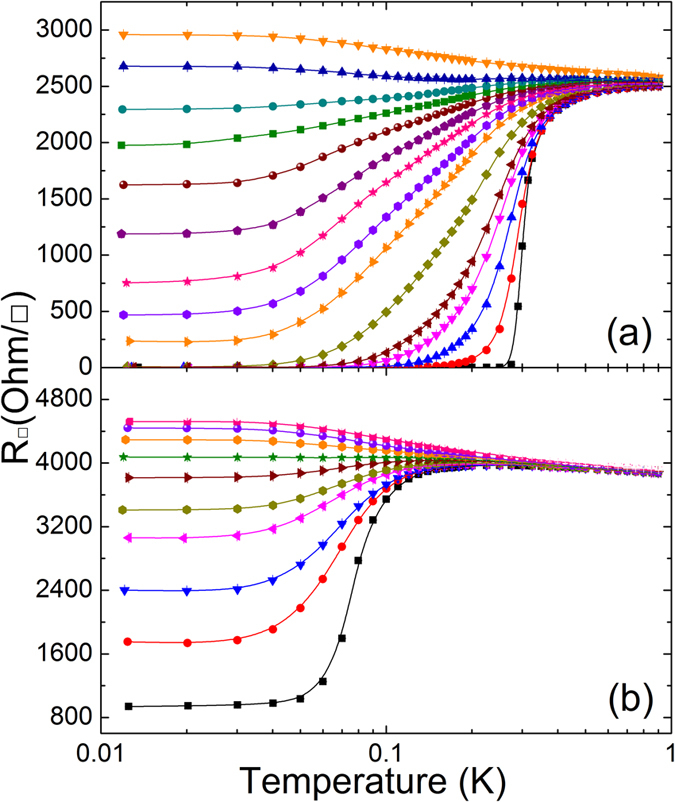
(**a**) Temperature dependence of sheet resistance under the magnetic field in the 4.5-nm superconducting sample, from the bottom, 0, 0.02, 0.05, 0.08, 0.1, 0.15, 0.2, 0.225, 0.25, 0.275, 0.3, 0.325, 0.35, 0.4, 0.6 T. (**b**) Temperature dependence of sheet resistance under the magnetic field in the 3.4-nm metallic sample, from the bottom, 0, 0.02, 0.03, 0.05, 0.06, 0.08, 0.1, 0.125, 0.2, 0.3 T. The data points are raw data and the solid curves are guides to the eye.

Figure 3(a) and (b) show the sheet resistance of Ta films with two different thicknesses – 4.5 nm and 3.4 nm, respectively – with various magnetic fields as a function of temperature. We find that the escalation of the magnetic field alters the nature of the transition dramatically. For instance, in the presence of a magnetic field of 0.2 T, the superconducting thin film with a 4.5-nm thickness is transformed to the metallic phase with finite saturated resistance at low temperatures. The field-induced metallic phase was found in a wide range of magnetic fields where the characteristic features were vastly consistent with the previous results reported by Yoon’s group^7^,^8^,^9^. With increasing magnetic field, the saturated resistance increases monotonically toward the normal-state resistance. The sheet resistance measured at B = 0.4 T exhibits negative d*R*/d*T* dependence, demonstrating an insulating phase. For the disorder-induced metallic sample, the saturated finite sheet resistance monotonically increases with increasing magnetic field and subsequently enters an insulating phase at a magnetic field higher than 0.1 T, as shown in Fig. 3(b).

**Figure 4:**
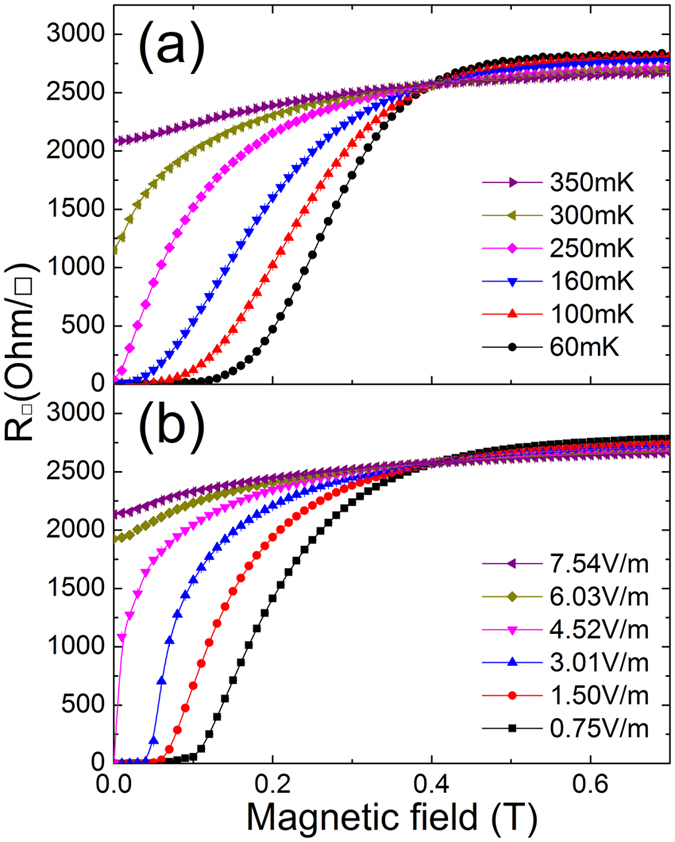
(**a**) Magnetic field dependence of sheet resistance at indicated temperatures in the 4.5-nm superconducting sample. (**b**) Magnetic field dependence of sheet resistance with various electric fields at T = 13 mK. The data points are raw data and the solid curves are guides to the eye.

The magneto-resistance curves for the 4.5-nm-thick superconducting sample measured at various temperatures are shown in Fig. 4(a). The sample was cooled to target temperatures without magnetic field, and the sheet resistance was measured with increasing magnetic field. The magneto-resistance isotherms cross at the characteristic value of magnetic field where the resistance is independent of temperature. The d*R*/d*T* is positive below this critical point and is negative above this point. This characteristic point can be defined as the critical field, *B*_*c*_, which divides the sample into an insulating phase and a superconducting phase. The disorder-induced metallic samples share essentially identical features with the superconducting samples. The critical field decreased systematically with decreasing film thickness, and no negative magneto-resistance region was found in any thickness range apart from the result reported for several thin films^41^,^42^,^43^,^44^,^45^. Instead of temperature, we measured the magneto-resistance curves for various electric fields at the base temperature. The resistance was measured at a certain target electric field as magnetic field increased. We observed approximately the same critical field, *B*_*c*_, in this set of measurements as that measured with the magneto-resistance isotherms. The magneto-resistance, as shown in the Fig. 4(b), is independent of the electric field at this critical point, *B*_*c*_. The sheet resistance increases when measured with increasing electric field below *B*_*c*_ and decreases above this value.

At the critical point of the magnetic-field-induced SI transition, the correlation length diverges as 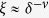, and the characteristic frequency vanishes as 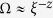, where 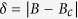, *ν* is the correlation length exponent and *z* is the dynamic critical exponent. Since the critical length fluctuation is the most important fluctuation near the transition, critical exponents *ν* and *z* are independent of materials and microscopic details when microscopic disorder is sufficiently homogeneous. The scaling behavior of a 2D disordered superconducting system near the critical field can be expressed by^1^,^26^


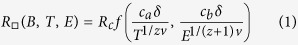


where *R*_*c*_ = *h/4e*^2^ is the universal sheet resistance at the critical field and *f*(*x*) is a universal scaling function. Exponents *ν* and *z* can be obtained independently by combining two scaling analysis of the electric-field-tuned measurements and the temperature-tuned measurements^26^. According to the previous electric field scaling in the 2D superconductor-insulator transition, the AC dynamic resistance was used for the electric field scaling. Instead, we used the DC resistance that was suggested in the Sondhi, *et al*.^1^ and also used in the previous electric field scaling for 2D metal-insulator transition^46^,^47^ and quantum hall effect^15^. It is notable that the scaling analysis with the AC dynamic resistance leads to the failure to obtain reasonable scaling exponents while the analysis with the DC resistance does not.

**Figure 5:**
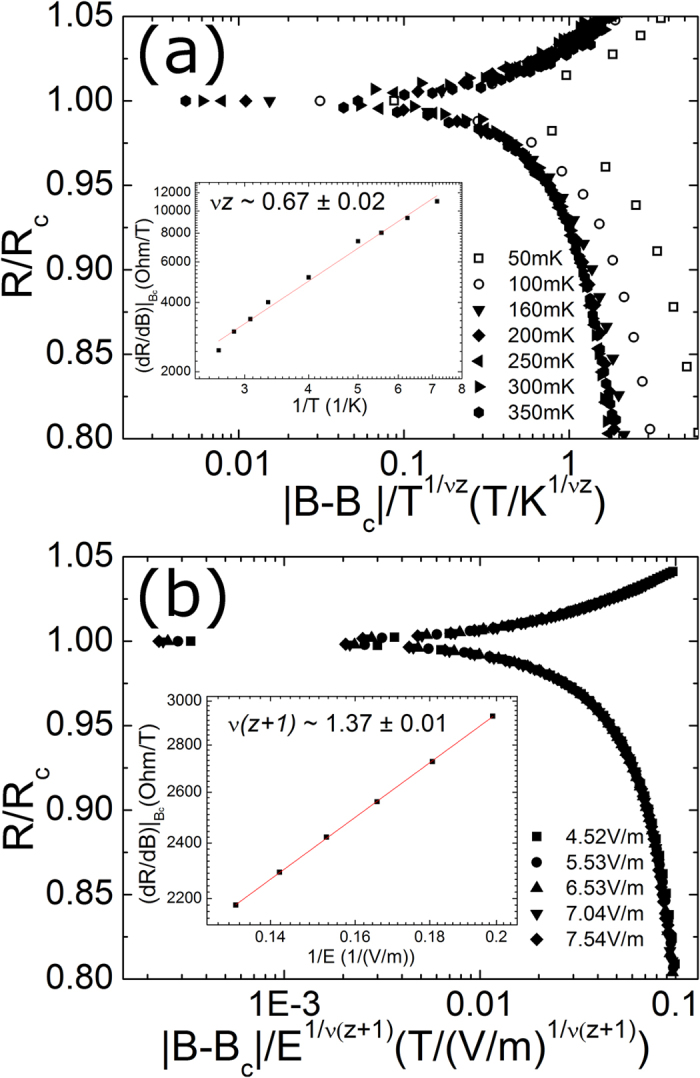
(**a**) Normalized resistance of the 4.5-nm superconducting sample as a function of the scaling variable 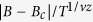 at indicated temperatures (for clarity, only seven temperatures are shown). Inset: The fitting of a power law to the inverse temperature dependence of d*R*/d*B* at *B*_*c*_. (**b**) Normalized resistance of the identical superconducting sample as a function of the scaling variable 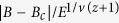 at indicated electric fields (for clarity, only five electric fields are shown). Inset: The fitting of a power law to the inverse electric field dependence of d*R*/d*B* at *B*_*c*_.

We utilized *B*_*c*_ determined from the magneto-resistance isotherms for the scaling analyses. In Fig. 5(a) and (b), the magneto-resistance isotherms of the 4.5-nm superconducting sample are plotted as a function of the scaling variable 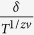 for the temperature scaling analysis and 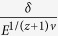 for the electric field scaling analysis. We determined the product *νz* by evaluating the mathematical relation 
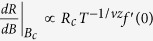
5. For instance, d*R*/d*B* at *B*_*c*_ is depicted as a function of the inverse temperature in the inset of Fig. 5(a), and the product *νz* is determined by evaluating the inverse slope of the log-log plot of 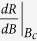
vs. 1/*T*. The critical exponent product is found to be 0.67 ± 0.02. Without applying any fitting parameter, the scaling function can be directly tested by substituting *B*_*c*_ and *νz* into the function. The resistance isotherms measured at temperatures higher than 0.25 K collapse onto a single curve, as shown in Fig. 5(a), which indicates that the scaling function is well obeyed. However, the resistance isotherms measured at low temperatures deviate from the scaling curve of the high-temperature isotherms, which suggests that the transition at low temperatures does not belong to the same universality class as the SI transition. We notice that a similar low-temperature deviation was found in MoGe superconducting films, and this deviation was ascribed to the coupling of the system to a dissipative bath^11^. The magneto-resistance was measured with various electric fields at T = 13 mK where no apparent change in resistance appears as a function of temperature. We determined the product *ν*(*z* + *1*) from the electric field scaling analysis using essentially the same procedure applied to the preceding temperature scaling analysis. The mathematical relation 
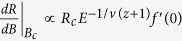
 was used to evaluate the product *ν*(*z* + *1*)^26^. As shown in the inset of Fig. 5(b), the inverse slope of 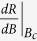 as a function of the inverse electric field (plotted in logarithmic scale) was used to find *ν*(*z* + *1*) = 1.37 ± 0.01. The scaling behavior is very well obeyed for sheet resistance isotherms plotted using the exponent product *ν*(*z* + *1*), as shown in Fig. 5(b). The critical exponents of the superconducting sample from both independent procedures are determined to be *ν*~0.69 ± 0.04 and *z*~0.98 ± 0.14. We noticed that the critical exponent *ν* is smaller than the theoretically predicted lower bound of *ν* ≥ 1 in the two-dimensional systems^6^. The exponent is essentially different from those of InO_*x*_ ref. 5 and MoGe^26^ and similar to the finding in the results for a-Bi^27^. The discrepancy in the scaling analyses can be related with the nature of disorder^26^. For example, our result is consistent with the universality class of the classical 3D XY model which is equivalent to two-dimensional systems without disorder. Numerical simulations for the Boson Hubbard model in a two-dimensional system without disorder also reveal a correlation length exponent of 0.7. In addition, the correlation length could be altered by disorder averaging, which might allow for *ν* less than 1 even in a disordered system^48^. We speculate that the discrepancies may be explained by the difference in the nature of the disorder between composite materials such as InO_*x*_ and MoGe, and monatomic materials such as a-Bi and Ta. For thin films comprised of monatomic materials, the disorder is solely controlled by film thickness, while disorder can be characterized in a more complicated way by the composition ratio in addition to the film thickness for composite materials. The abundant microstructures induced by various atomic compositions and annealing processes may augment the random disorder in those thin films.

**Figure 6:**
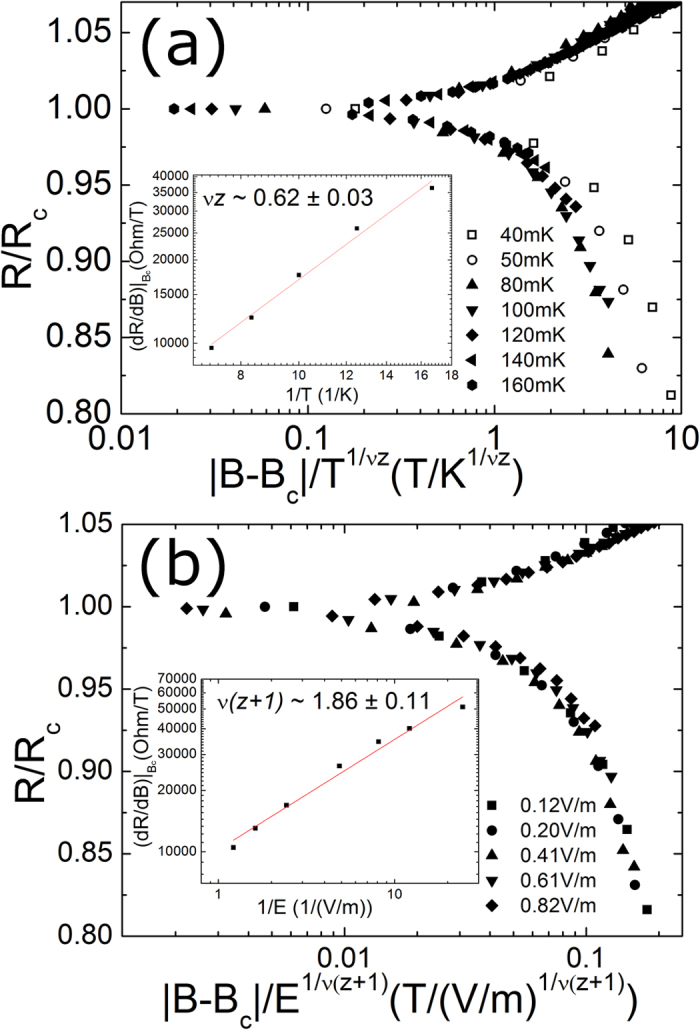
(**a**) Normalized resistance of the 3.4-nm metallic sample as a function of the scaling variable 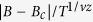 at indicated temperatures (for clarity, only seven temperatures are shown). Inset: The fitting of a power law to the inverse temperature dependence of d*R*/d*B* at *B*_*c*_. (**b**) Normalized resistance of the identical metallic sample as a function of the scaling variable 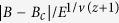 at indicated electric fields (for clarity, only five electric fields are shown). Inset: The fitting a power law to the inverse electric field dependence of d*R*/d*B* at B_c_.

The temperature scaling result for the disorder-induced metallic sample which is 3.4nm-thick sample is shown in Fig. 6(a) and the electric field scaling conducted at 13 mK in Fig. 6(b). We find that the scaling data are visually well collapsed with the same scaling function used in the superconducting sample. Furthermore, the low-temperature deviation from the main scaling curves in the metallic sample resembles that in the superconducting sample. The critical exponent product *νz* for the metallic sample is found to be 0.62 ± 0.03 which is not very different from that obtained in the superconducting sample. However, the product of *ν*(*z* + *1*) determined from the electric-field-tuned analysis is found to be about 1.86 ± 0.11, indicating a clear discrepancy from that of the superconducting sample. From the combination of the two measurements, we obtained *ν*~1.23 ± 0.20 and *z*~0.52 ± 0.16 which differ substantially from theoretical predictions and previous experimental results. The significant discrepancy in the dynamic critical exponent for the disorder-induced metallic samples implies that the electric field scaling may be invalid and the consequence of the collapsed data may be associated with an extrinsic effect such as electron heating^1^.

**Figure 7:**
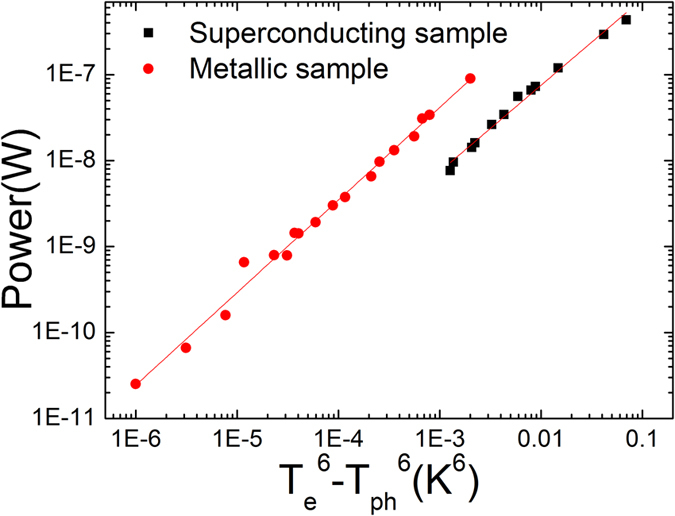
The (*T*_*e*_^6^−*T*_*ph*_^6^) dependence of the dissipated power. The fitted slops are 1 in the both samples which means the linear dependence of the power and (*T*_*e*_^6^−*T*_*ph*_^6^).

The validity of electric field scaling is discussed in the aspect of hot-electron effect^1^,^37^. The joule heating by the electric field is inevitable and weak electron-phonon coupling cannot dissipate the heating power *P* sufficiently. The produced power leads to the temperature gradient with the relation of 
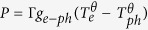
 where *Γ* is an active volume of sample, *g*_*e−ph*_ electron-phonon coupling constant, *T*_*e*_ temperature of hot electrons, *T*_*ph*_ temperature of phonons, and *θ* to be between 3.4 and 6 depending on materials^49^,^50^. According to the hot-electron effect, the electric field scaling could be converted to a ‘real’ electron temperature scaling because the electric field scaling could be dominated by the hot-electron effect rather than the intrinsic nonlinear electric field effect when *θ* is higher than 4. We measured the temperature dependence of the film resistance with a range of various bias currents to determine the *θ* value. Figure 7 shows the dissipated power as function of the *T*_*e*_^6^−*T*_*ph*_^6^ in the 5.1-nm superconducting and 3.35-nm metallic samples, indicating the best fitting with *θ* = 6. The result shows very good agreement with the previous report^50^. The high *θ* value of Ta thin films suggests that the failure of the electric field scaling can be attributed to the hot electron effects in the disorder-induced metallic films. Accordingly, the collapsed data can be the reflection of the temperature scaling due to the elevated electron temperature with joule heating. On the other hand, it is notable that the hot electron effect is not dominant in the superconducting films although the *θ* value is measured to be approximately 6 as well. We speculate that the heating effect is not relevant in the superconducting films far below the transition since the small density of quasi particle excitations or fermionic electrons is insufficient to produce heating. The joule heating can be suppressed vastly due to the existence of condensed bosons so that the electric field scaling is valid up to the sufficiently high electric field.

**Figure 8:**
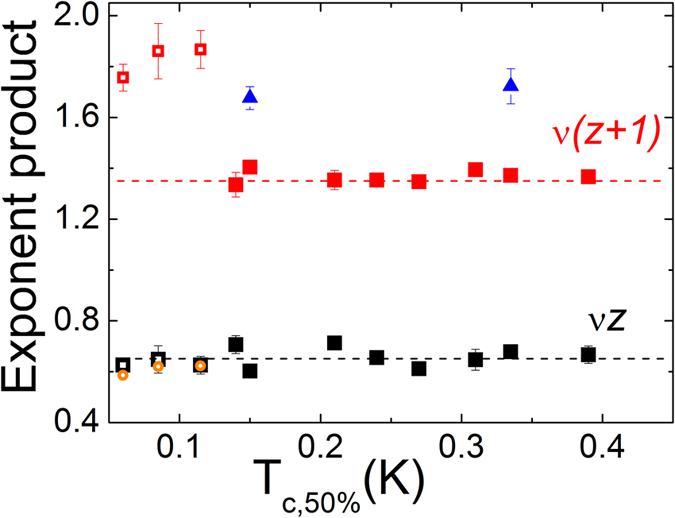
Transition temperature dependence of critical exponent product *νz* (black symbols) and *ν*(*z* + *1*) (red symbols and blue symbols) for a wide range of disorders. Solid symbols are for the superconducting samples and open symbols are for the metallic samples. Dashed lines are guides to the eyes for representative values. Blue symbols are critical exponent product *ν*(*z* + *1*) at high temperature and orange symbols are critical exponent product *νz* of ‘real’ electron temperature scaling analysis determined by electric field scaling exponent product.

The crucial difference between these two samples were clearly shown in the Fig. 8 where the product of critical exponents *νz* (black symbols) and *ν*(*z* + *1*) (red and blue symbols) as a function of the critical temperature of the Ta films with various thicknesses were depicted. The solid symbols are for the superconducting samples which have *T_c_* higher than 0.13 K and the open symbols are for the metallic samples that show transition below 0.13 K. Besides, the product *ν*(*z* + *1*) results of two superconducting samples measured at high temperatures were depicted in blue triangles in comparison to the red squares measured at the base temperature. Pronounced transition in the critical exponent product *ν*(*z* + *1*) from the metallic to the superconducting samples appears as shown in Fig. 8. One may speculate that the strong deviation from the expected values may be associated with the fact that the scaling analysis for the disorder induced metallic films could be different from that of the standard SI transition. However, we found the deviation could be ascribed to the strong evidence of electron heating. First, we reconstructed electric filed scaling analysis by mapping the electric power to the electron temperature as described in the ref. 37. The adjusted scaling result is plotted in Fig. 8 with open orange symbols. We found the electric field scaling could be collapsed by the ‘real’ electron temperature scaling variables. The exponent product *νz* with ‘real’ electron temperature scaling analysis provided from the electric field scaling analysis is about 0.62 which shows a good agreement with critical exponent product of the temperature scaling analysis. Second, we performed the electric field scaling at the elevated temperatures where the superconducting samples show about 50% of the normal resistance value. One can consider the introduction of dissipation due to the finite resistance and, consequently, joule heating in the sample. As shown in the Fig. 8 with blue triangles, the product *ν*(*z* + *1*) measured with hot electrons shows dramatic shift from ~1.36 to ~1.7. These remarkable shifts demonstrate that the electric field scaling analysis for the superconducting samples can be strongly altered by the joule heating, which could be responsible for the previous measurements of high *θ* as well.

Although the electric field scaling analysis in the disorder-induced metallic sample is ascribed to the heating effect, the temperature scaling analysis provides useful information. The critical exponent *ν* of disorder-induced metallic samples is found to be approximately 0.62 assuming that *z* = 1, which shows good agreement with the values of superconducting samples. No clear difference between the superconducting and disorder-induced metallic samples may suggest that the SI scaling analysis is not sensitive to the existence of disorder or no fundamental difference exists between two phases. One may argue that a new scaling analysis is necessary to understand the metal–insulator (MI) transition scaling^19^ on the assumption that standard SI scaling cannot be applied to the metallic ground state in Ta thin films. We found that the suggested MI scaling function of the ref. 19 was insensitive to the choice of critical exponents due to the diverging nature at the critical point. Despite of the well-collapsed scaling curves over wide temperature ranges, we cannot determine the critical exponents. One may also suggest that the metallic phase is only possible at low temperature by a sample connected via a dissipative fermionic bath. Accordingly, the scaling analysis near *T*_*c*_ cannot probe the low temperature metallic phase but the SI transition at high temperatures.

## Conclusion

The scaling analysis for Ta thin films with a wide range of disorders is performed. Critical exponents *ν* and *z* are obtained independently by performing the temperature-tuned and electric-field-tuned scaling analysis. The superconducting samples reveal the well-obeyed dynamic critical exponent of approximately unity, whereas the apparent deviation of critical exponent *ν* from the theoretical predictions and from the previous experimental results was found. In addition, we find that the critical exponents obtained from the disorder-induced metallic samples – which are clearly inconsistent with theoretical predictions – show a marked discrepancy from the exponents obtained for the superconducting samples. The seemingly apparent transition between these two types of samples is the consequence of the joule heating of electron and not the indication of the disorder-induced metallic film being the different universality class from that of two-dimensional superconducting films.

## Methods

Ta thin films were fabricated by a DC sputtering technique. The thickness of a sample was determined by a quartz microbalance during sputtering and was confirmed by measurement with an AFM after sputtering. The sample has a Hall-bar shape (1 mm in width and 5 mm in length) for standard four-probe lock-in amplifier measurements, I_ac_ = 0.1~1 nA and f = 13.33 Hz. The magnetic fields are applied perpendicular to the sample plane and the electric fields are applied parallel to the sample plane. For low noise measurements, all measurement lines utilized low temperature coaxial cables, pi filters, and two stage RC filters to avoid rf noises. Low-temperature measurements are performed with home-made cryo-free dilution refrigerators and a conventional cryogen dilution refrigerator (Oxford Kelvinox).

## Additional Information

**How to cite this article:** Park, S. *et al*. Scaling analysis of field-tuned superconductor–insulator transition in two-dimensional tantalum thin films. *Sci. Rep.* **7**, 42969; doi: 10.1038/srep42969 (2017).

**Publisher's note:** Springer Nature remains neutral with regard to jurisdictional claims in published maps and institutional affiliations.
